# Proteinuria impacts patient survival differentially based on clinical setting: A retrospective cross-sectional analysis of cohorts from a single health system: Retrospective cohort study

**DOI:** 10.1016/j.amsu.2019.07.029

**Published:** 2019-08-01

**Authors:** Adam Bezinque, Jessica Parker, Stephen K. Babitz, Sabrina L. Noyes, Susie Hu, Brian R. Lane

**Affiliations:** a Michigan State University College of Osteopathic Medicine, East Lansing, MI, 48824, USA; b Spectrum Health Hospital System, Grand Rapids, MI, 49503, USA; c Warren Alpert Medical School of Brown University, Division of Kidney Disease and Hypertension, Providence, RI, 02903, USA; d Michigan State University College of Human Medicine, Grand Rapids, MI, 49503, USA

**Keywords:** Urinalysis, Proteinuria, Survival, Albuminuria, Chronic kidney disease, Glomerular filtration rate

## Abstract

**Background:**

Chronic kidney disease (CKD) staging is improved by adding proteinuria to glomerular filtration rate (GFR). While proteinuria independently predicts CKD progression and mortality, the clinical setting of proteinuria determination has not been well-studied previously. The objective of our study is to determine whether clinical setting differentially impacts survival outcomes.

**Methods:**

Kaplan-Meier and Cox proportional hazards analyses of overall survival were performed retrospectively for cohorts of outpatients (n = 22,918), emergency patients (n = 16,861), and inpatients (n = 12,304) subjected to urinalysis (UA) at a single health system in 2010. GFR (G1-G5) and proteinuria (A1:<30 mg, A2:30–300 mg, A3:>300 mg) were classified under Kidney Disease: Improving Global Outcomes (KDIGO) guidelines.

**Results:**

GFR and proteinuria levels varied more in inpatients than in emergency and outpatients. For each clinical population, survival significantly decreased with increasing proteinuria (A1>A2>A3, p < 0.05 for each comparison). The effect of proteinuria on survival differed by clinical setting, with statistical differences in all categories other than A3 in outpatients and emergency patients (p = 0.98). The strongest predictors of mortality were cancer diagnosis (HR: 3.07, p < 0.0001) and very-high KDIGO classification (HR: 2.01, p < 0.0001). Limitations include the retrospective observational study design and single health system analysis.

**Conclusions:**

The value of UA to screen for proteinuria in each clinical setting is evident, but the impact of A2 and A3 level proteinuria on survival varies depending on the clinical scenario in which the determination was made. The clinical setting of proteinuria measurement should be factored into both patient care and clinical research activities.

## Highlights

• Proteinuria and GFR independently predict survival in inpatients, ED and outpatients.
• The effect of proteinuria on survival differs by clinical setting.
• Hospitalized patients have lower survival rates than emergency and outpatients.
• Clinical setting of proteinuria should be considered in patient care and research.

## 1 Introduction

Identification of chronic kidney disease (CKD) is critical to prevent end stage renal disease (ESRD) and achieve more favorable survival outcomes [1]. Although initial guidelines classified CKD solely based on estimated glomerular filtration rate (GFR), the National Kidney Foundation in the K/DOQI guidelines and PARADE study indicates that both GFR and proteinuria are important factors in CKD risk stratification [2,3]. CKD classification based on GFR, proteinuria, and etiology accurately predicts progression of CKD and mortality in diverse patient populations [[4], [5], [6]].

In prior studies, CKD groupings have been made utilizing proteinuria determinations from urinalysis (UA), 24-h urine collection, albumin-to-creatinine ratio (ACR), or unspecified methodology [7,8]. In addition, classification of the clinical setting in which proteinuria was detected has been routinely omitted. Higher levels of proteinuria on UA are associated with increased risk of death and progression to kidney failure at a given level of GFR, indicating the importance of this marker as a sign of significant kidney damage [4,9,10]. For example, the risk of death doubled in patients with stage 2 CKD having proteinuria, compared to the stage 2 CKD population without proteinuria [11]. Dipstick UA is a widely accepted test performed commonly in a variety of clinical settings. UA is reliably diagnostic of proteinuria with high sensitivity and specificity; however, a very low percentage of positive dipsticks are indicative of serious urinary tract disorders [[12], [13], [14], [15]]. Screening of the general population for proteinuria with UA is not common practice, although it is recommended for high-risk populations such as those diagnosed with hypertension, diabetes, and/or greater than 60 years of age [[16], [17], [18]]. A greater understanding of survival in high-risk patient populations will provide guidance into the overall clinical course and management of CKD.

Although proteinuria is clearly a predictor of CKD progression and mortality, the clinical setting in which proteinuria was determined has not been well documented or studied and may significantly impact CKD classification and survival. A high prevalence of proteinuria can be detected in the inpatient setting, but this may be attributable to acute kidney injury or failure, rather than CKD [19]. In the current study, we evaluate the survival of a large number of patients having UA in outpatient, inpatient, and emergency settings according to proteinuria classification.

## 2 Materials and methods

### 2.1 Patient populations

Patients having both UA and serum creatinine determination in the same 24-h period were identified retrospectively within Spectrum Health Hospital System records during calendar year 2010. Patients were then separated according to clinical setting in which these laboratory studies were performed: outpatient (OP) medical practices: 22,918, emergency department (ED) patients: 16,861, and hospitalized inpatients (IP): 12,304. Follow-up on each unique patient was obtained directly from the electronic medical record. Clinical and demographic data for patients in each cohort were recorded in a database with only de-identified data. The Spectrum Health Institutional Review Board approved the study, which has been registered at ResearchRegistry5017. This research is fully compliant with the STROCSS criteria [20].

### 2.2 Urine analysis

Quantification of proteinuria was performed with a Clinitek urine analyzer (Siemens Medical Solutions USA, Inc., Malvern, PA). Proteinuria was classified according to KDIGO guidelines, as A1 when ‘negative’, ‘trace’, 15 or 25 mg/g; A2 when 1+ (30 mg/g), 2+ (100 mg/g), or 3+ (300 mg/g); and A3 when 4+ (>300 mg/g).

### 2.3 Statistical analysis

Demographic analyses were done using a Kruskal Wallis analysis for quantitative data and a chi-square analysis for qualitative data. Quantitative data was expressed as median [quartile 1, quartile 3] and qualitative data was expressed as frequency (percent). Survival curves and estimates were created assessing survival probability at 2, 4, and 6 years after measurement. A1, A2, and A3 proteinuria groups were plotted accordingly. Log-Rank tests were performed with a Tukey-Kramer adjustment for pairwise comparisons to determine statistical significance with p < 0.05. Cox proportional hazards models were run utilizing either KDIGO classification or GFR group and proteinuria group along with age, race, gender, and comorbidities. Statistical analyses were generated using SAS (SAS Enterprise Guide software, Version 7.1, SAS Institute Inc, Cary, NC).

## 3 Results

### 3.1 Impact of clinical setting on proteinuria and glomerular filtration rate

**Fig. 1:**
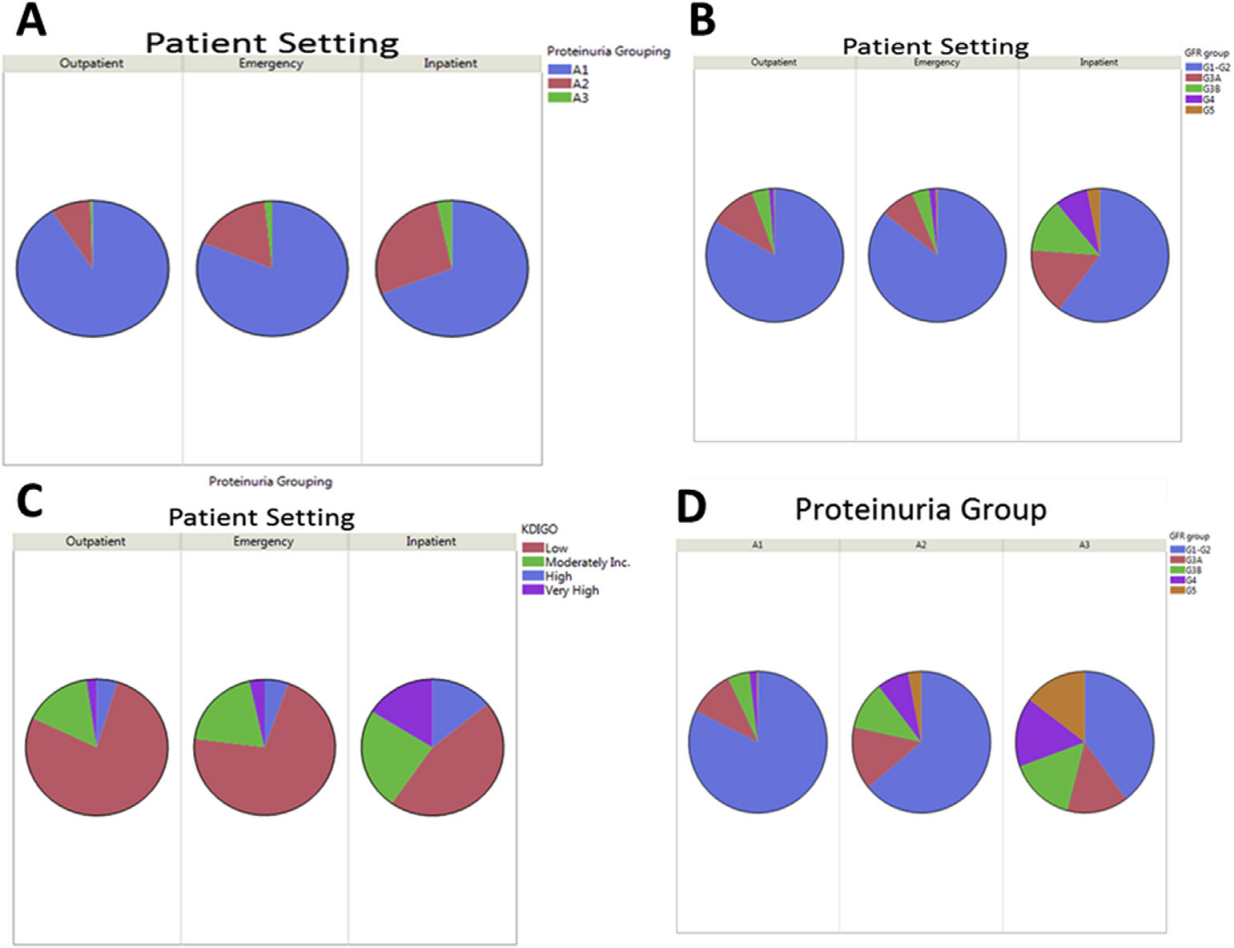
Associations between proteinuria, GFR, KDIGO risk group, and clinical setting in which UA was performed. (A) Proteinuria Group, (B) GFR Group, and (C) KDIGO Risk Stratification by Patient Setting. (D) GFR Group Distribution by proteinuria group.

Multiple differences were identified in the UA samples obtained from patients in the OP, ED, and IP settings (Table 1). Negative dipstick as defined by no positive blood, glucose, leukocyte esterase, nitrite, ketone, urobilinogen, and bilirubin was 70.9% for outpatients, 50.6% for ED patients, and 44.0% for inpatients. The proportion of patients reported to have no significant microscopic UA findings was 46.2% for outpatients, 27.1% for ED patients, and 32.1% for inpatients. The proportion of patients with positive findings, and distribution of these findings, was also significantly different between the three cohorts (Table S1). Proteinuria was present in 17.3% of patients overall, with proportions of 9.0%, 18.7%, and 31.0% in the OP, ED, and IP groups, respectively (Table 1, Fig. 1A). Median creatinine was also significantly higher for IP (median: 0.98 mg/dL, IQR: 0.77–1.35, p < 0.0001), than both OP (median: 0.88, IQR: 0.74–1.04) and ED patients (median: 0.80, IQR: 0.67–0.98). This corresponded to a shift in distribution of GFR classification away from G1-G2 in OP and ED patients, with greater proportions of G3a, G3b, G4, and G5 groups in IP (p < 0.0001) (Fig. 1B). Higher GFR group was associated with the presence of proteinuria, resulting in a greater proportion of patients with moderately increased, high, and very high CKD risk (according to KDIGO classification) for IP compared with ED and OP (p < 0.0001, Fig. 1C and D).

**Table 1:** Urinalysis data according to patient setting group.

	All Patients (n = 52083)	Outpatient (n = 22918)	Emergency Department (n = 16861)	Inpatient (n = 12304)	p
Median Age, years (IQR)	55 (39, 70)	57 (46, 68)	41 (28, 60)	66 (50, 80)	<0.0001
Male	21081 (40.5%)	10435 (45.5%)	5174 (30.7%)	5472 (44.5%)	<0.0001
African-American	4879 (9.4%)	1482 (6.5%)	2372 (14.1%)	1025 (8.3%)	<0.0001
Negative Urinalysis Findings					<0.0001
Dipstick	30126 (57.8%)	16180 (70.9%)	8532 (50.6%)	5414 (44.0%)	
Microscopic	19107 (36.7%)	10588 (46.2%)	4569 (27.1%)	3950 (32.1%)	
Proteinuria group					<0.0001
A1(<30, negative, trace)	43055 (82.7%)	20856 (91.0%)	13715 (81.3%)	8484 (69.0%)	
A2 (30–300)	8220 (15.8%)	1942 (8.5%)	2861 (17.0%)	3417 (27.8%)	
A3 (>300)	808 (1.5%)	120 (0.5%)	285 (1.7%)	403 (3.2%)	
Median Creatinine, mg/dL (IQR)	0.87 (0.72, 1.06)	0.88 (0.74, 1.04)	0.80 (0.67, 0.98)	0.98 (0.77, 1.35)	<0.0001
GFR Group					<0.0001
G1-G2 (>60)	41007 (78.8%)	19129 (83.5%)	14480 (85.9%)	7398 (60.1%)	
G3a (45–60)	5873 (11.3%)	2537 (11.1%)	1367 (8.1%)	1969 (16.0%)	
G3b (30–45)	3233 (6.2%)	947 (4.1%)	682 (4.0%)	1604 (13.0%)	
G4 (15–30)	1479 (2.8%)	260 (1.1%)	264 (1.6%)	955 (7.8%)	
G5 (<30)	483 (0.92%)	42 (0.2%)	67 (0.4%)	374 (3.0%)	
KDIGO Risk Classification					<0.0001
Low	35429 (68.0%)	17738 (77.4%)	12070 (71.6%)	5621 (45.7%)	
Moderately Increased	9828 (18.9%)	3574 (15.6%)	3291 (19.5%)	2963 (24.1%)	
High	3707 (7.1%)	1069 (4.7%)	911 (5.4%)	1727 (14.0%)	
Very High	3111 (6.0%)	534 (2.3%)	588 (3.5%)	1989 (16.2%)	

IQR, interquartile range; GFR, glomerular filtration rate; KDIGO, kidney disease: improving global outcomes.

### 3.2 Overall survival is differentially affected by proteinuria according to clinical setting

**Fig. 2:**
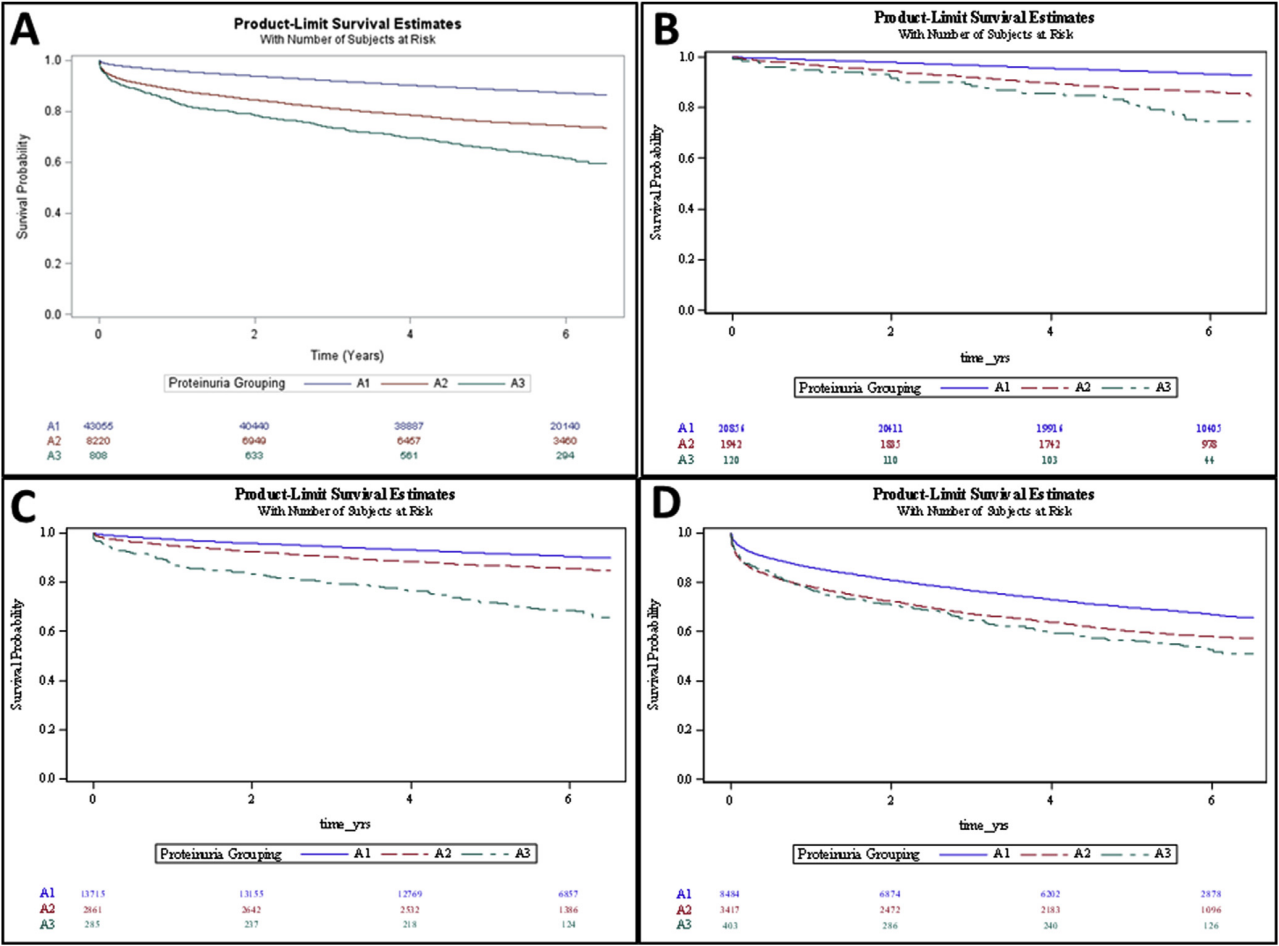
Kaplan-Meier overall survival curves stratified by proteinuria groups (log rank p < 0.0001). (A) All Patients. (B) Outpatient Population. (C) Emergency Population. (D) Inpatient Population.

**Fig. 3:**
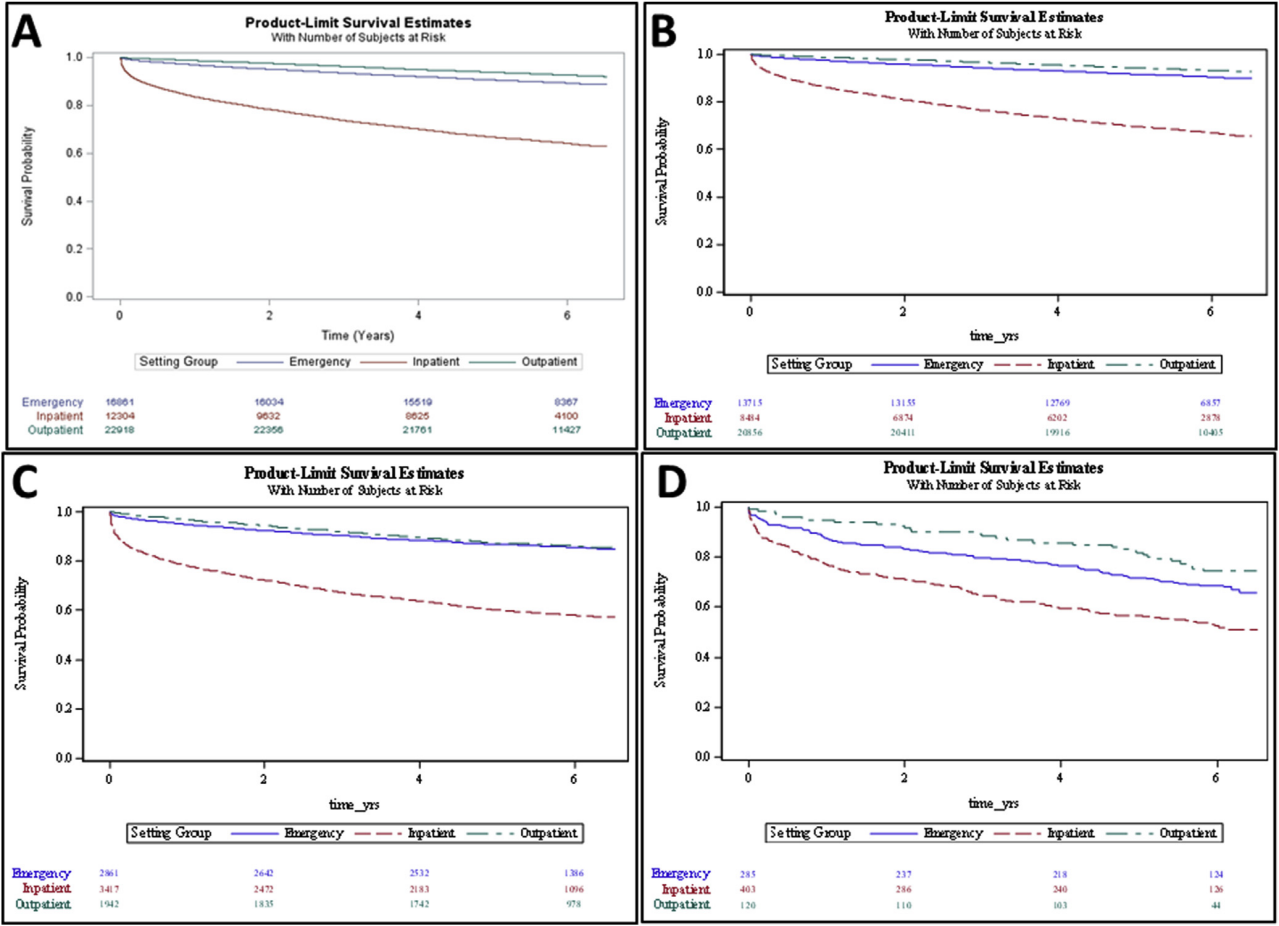
Kaplan-Meier overall survival curves stratified by proteinuria groups (log rank p < 0.0001) (A) All Patients. (B) A1 Proteinuria group. (C) A2 Proteinuria group. (D) A3 Proteinuria group.

The six-year overall survival rate of patients with A1 proteinuria group on UA was 93.3% for OP, 90.5% for ED, and 67.0% for IP (Table 2). For A2 proteinuria group on UA, the survival rate of patients was 86.1% for OP, 85.5% for ED, and 58.0% for IP. For A3 proteinuria group on UA, survival was 75.0% for OP, 67.7% for ED, and 52.3% for IP (Fig. 2). For each patient population, survival was significantly decreased in the higher proteinuria group relative to lower proteinuria group (A1>A2>A3) at p < 0.0001, except for ED A2>A3 at p = 0.0086 (Table S2). When comparing different patient settings within each proteinuria group, all comparisons were significant at p < 0.0001 (Table S3), with the exception of A2 (OP > ED, p = 0.0130), and A3 for OP and ED groups, which were not significantly different (p = 0.98) (see Fig. 3).

**Table 2:** Overall survival at 6 years according to proteinuria group and clinical setting.

Patient Group	Proteinuria Group	Total (n)	Deceased (n)	Surviving (n)	Survival (%)
Outpatient	A1	20856	1400	19456	93.29
	A2	1942	270	1672	86.10
	A3	120	30	90	75.00
	Total	22918	1700	21218	92.58
Emergency	A1	13715	1308	12407	90.46
	A2	2861	415	2446	85.49
	A3	285	92	193	67.72
	Total	16861	1815	15046	89.24
Inpatient	A1	8484	2799	5685	67.01
	A2	3417	1436	1981	57.97
	A3	403	192	211	52.36
	Total	12304	4427	7877	64.02
Total		52083	7942	44141	84.75

To further investigate the interaction between the extent of proteinuria and the clinical setting in which it was determined, we performed multivariable analyses of survival accounting for patient demographics and comorbidities. In Cox proportional hazard models, the only variables that were not significantly associated with survival were peripheral vascular disease and race (Table 3, p < 0.0001 for all significant variables). For KDIGO classification, GFR group and proteinuria group, the hazard ratio (HR) increased with ascending group or classification. Cancer had the highest HR among comorbidities in both the KDIGO based model (3.07, 95%CI: 2.84–3.32) and the GFR/proteinuria based model (3.05, 95%CI: 2.82–3.30). Cox analysis also confirmed the IP population to be the most at-risk population in both the KDIGO-based model [HR:1.61 (95% CI: 1.52–1.70)] and the GFR/proteinuria based model [HR: 1.59 (95% CI: 1.50–1.68)].

**Table 3:** Cox proportional hazards regression analysis with mortality as the dependent variable, with either KDIGO class or both proteinuria and GFR groups included as independent variables.

Variable	KDIGO Used		Proteinuria & GFR Used	
	Hazard Ratio (95% CI)	p-value	Hazard Ratio (95% CI)	p-value
Age	1.059 (1.057–1.060)	<0.0001	1.060 (1.058–1.062)	<0.0001
Gender (Female vs. Male)	1.26 (1.21, 1.32)	<0.0001	1.25 (1.20, 1.31)	<0.0001
Race (African American vs. Other)^f^	–	0.6235	–	0.3231
Hypertension	0.85 (0.81–0.90)	<0.0001	0.86 (0.82–0.90)	<0.0001
Diabetes	1.31 (1.24–1.39)	<0.0001	1.30 (1.23–1.38)	<0.0001
CAD/MI	1.25 (1.16–1.35)	<0.0001	1.24 (1.15–1.34)	<0.0001
PVD^f^	–	0.0748	–	0.0582
Cancer	3.07 (2.84–3.32)	<0.0001	3.05 (2.82–3.30)	<0.0001
Patient Setting^a^				
Inpatient	1.61 (1.52–1.70)	<0.0001	1.59 (1.50–1.68)	<0.0001
Outpatient	0.47 (0.44–0.50)	<0.0001	0.47 (0.44–0.50)	<0.0001
KDIGO Class^b^			NA^c^	NA
Class 2 (Moderately-Increased)	1.32 (1.24–1.40)	<0.0001		
Class 3 (High)	1.58 (1.47–1.69)	<0.0001		
Class 4 (Very High)	2.01 (1.88–2.15)	<0.0001		
GFR Group^d^	NA^c^	NA		
G3a			1.14 (1.07–1.21)	<0.0001
G3b			1.44 (1.34–1.53)	<0.0001
G4			1.62 (1.49–1.76)	<0.0001
G5			1.98 (1.73–2.25)	<0.0001
Proteinuria Group^e^	NA^c^	NA		
A2			1.36 (1.29–1.43)	<0.0001
A3			1.70 (1.51–1.90)	<0.0001

KDIGO, kidney disease: improving global outcomes; GFR, Glomerular filtration rate; PVD, peripheral vascular disease; CAD, coronary artery disease; MI, Myocardial infarction.

a Reference group = Emergency Department.

b Reference group = KDIGO Class 1 (Low).

c Variable not included in this analysis.

d Reference group = GFR group G1/G2.

e Reference group = Proteinuria group A1.

f Hazard ratios were not calculated and variable was not included in the model, due to failure to enter the model at p < 0.05.

## 4 Discussion

The most recent KDIGO schema for CKD classification incorporates etiology, GFR, and proteinuria status [6]. These guidelines are clear about GFR measurements, which must include at least two readings >90 days apart, but do not clearly state the timing or setting for proteinuria determination. Although bloodwork for CKD screening (including sCr and eGFR) is routinely performed in U.S. primary care settings, dipstick UA is not, primarily because cost effectiveness has not been confirmed [21]. One concern is that broad application of dipstick UA has the potential to increase unnecessary additional evaluations due to the propensity of false-positive results. In one study, significant urinary tract disorders were detected in less than 1.5% of asymptomatic adults with screen-identified proteinuria [12]. In higher risk populations, such as patients who have been hospitalized, routine UA can prove beneficial [22,23]. In addition to identification of proteinuria, UA is useful for the detection of pyuria, hematuria, excreted glucose, erythrocytes, and biomarkers for liver disease, and other related comorbidities [24]. In other countries, such as Japan, where the relative prevalence of proteinuria in the population is higher, universal screening is common practice [25].

Proteinuria has several etiologies including glomerular, tubular, overflow, and transient. Glomerular proteinuria is the most common etiology, reflecting an intrinsic insult to the renal parenchyma with albumin as the main excretory product [26]. Patients in the Framingham study, which are felt to approximate the general U.S. population, were found to have a three-fold increase in mortality if they were positive for proteinuria [27,28]. Although the Framingham study was not focused on renal outcomes, the risk of progression to kidney failure was independently increased in patients with higher levels of proteinuria [10,29]. UA is shown to be a reliable method to detect proteinuria and diagnose CKD stage 2 [11]. While microalbuminuria has been suggested to be predictive of negative outcomes in older critically ill patients, to the best of our knowledge, it has not yet been assessed whether evaluation in broader patient settings influences survival [30].

In our study, increasing levels of proteinuria were associated with decreased overall survival within patients undergoing UA in each clinical situation, with ED patients having the greatest disparity from A1 to A3 (90%–68% at 6 years, respectively). These findings are consistent with the present literature and advance the present understanding of the role of proteinuria as an indicator of faster progression to kidney failure and an increased mortality [10,29,31].

Those with inpatient UA measurement had the lowest survival in all proteinuria groups (67%, 58%, and 52% for A1, A2, and A3, respectively). Outpatients were shown to have the best survival with 6-year rates of 93%, 86%, and 75% for A1, A2, and A3, respectively. These data suggest that UA is a strong predictor of survival in each of these clinical settings, but behaves differently in each. When urinalyses are to be used for determination of proteinuria related to CKD, these data suggest that inpatient and Ed settings are not equivalent to the outpatient setting. If broad screening is to be performed, these data suggest that urinalyses only from the outpatient setting be used. Alternatively, if screening is felt only to be of benefit in high-risk patients, UA would be of highest yield for hospitalized patients and those with a cancer diagnosis, high CKD risk, and/or impaired renal function, as those groups of patients had the highest hazard ratios for all-cause mortality. Screening these patients may make them eligible for appropriate countermeasures to prolong survival.

Our results demonstrate that depending on the setting in which the UA was performed, long-term mortality will vary. While all three of our cohorts revealed an increase in mortality with rising proteinuria, hospitalized patients had the worst prognosis. Even hospitalized patients with no proteinuria exhibited greater short and long term mortality when compared with outpatients and ED patients. When assessing a patient's status, it is not enough to just consider lab results but to also take into account the overall clinical picture, which includes the risk as determined by the clinical setting. When conducting future retrospective studies or designing clinical trials, it is important to take into account that hospitalized patients represent a population that are inherently predisposed to both short- and long-term mortality.

Our study has several important limitations, including those occurring in any retrospective, observational analysis of a large patient cohort study. The indications for UA in each setting were subject to different selection biases. We were unable to assess medication usage within this dataset. Additionally, the West Michigan patient population may not be representative of more racially and ethnically diverse populations that have higher incidence of CKD. Validation in health care systems in other geographical locations will be necessary to corroborate our findings. Due to the large volume of UA and serum creatinine samples included, pre-existing CKD diagnosis could not be analyzed as it was not routinely recorded. The relationship between pre-existing CKD status and patient population settings is of interest for additional analysis. While our survival data does provide a longitudinal perspective for mortality outcomes, we were unable to track the corresponding longitudinal proteinuria and ACR data. Another limitation is variability in treatment between different settings, which could be accounted for in a longitudinal study with more detailed patient follow up.

Proteinuria is a strong predictor of mortality in all clinical settings. Hospitalized patients have a lower survival rate than outpatients and ED patients in all proteinuria groups. The value of UA in these three patient settings as a screening tool for proteinuria and other conditions appears high, as a high proportion ofpatients have proteinuria and other abnormalities on clinically-determined UA. Classification of CKD risk according to GFR, amount and setting of proteinuria, and cause, may better identify patients at increased risk for mortality so that appropriate management and nephrology referral can be arranged.

## Provenance and peer review

Not commissioned internally reviewed.

## Ethical approval

The Spectrum Health Institutional Review Board approved the study (IRB #2015–076)

## Sources of funding

Betz Family Endowment for Cancer Research & Spectrum Health Foundation.

## Author contribution

AB – data analysis, writing.

JP – data collection, statistics.

SB – data analysis, writing, statistics.

SN – data analysis, writing.

SH – manuscript writing.

BL – study design, writing, data analysis, supervision.

## Conflicts of interest

The authors have no conflicts of interest.

## Research registration number

www.researchregistry.com.

Researchregistry5017.

## Guarantor

Dr. Brian Lane.
