# Supplementary material for: Proteinuria impacts patient survival differentially based on clinical setting: A retrospective cross-sectional analysis of cohorts from a single health system: Retrospective cohort study
Source: Ann Med Surg (Lond). 2019 Aug 1;45:120–6. doi: 10.1016/j.amsu.2019.07.029 (PMC6702410; doi:10.1016/j.amsu.2019.07.029)
Supplement: Multimedia component 2 [file mmc2.docx]

**Table S1.** Additional data from urinalysis for all patients.

|  | **All Patients** (n=52083)  (n, % unless indicated otherwise) | **Chi-Square**  **P-Value**  (by Patient Setting) |
| --- | --- | --- |
| Proteinuria group  A1 (<30, negative, trace)  A2 (30-300)  A3 (>300) | 43055 (82.7%)  8220 (15.8%)  808 (1.5%) | <0.0001 |
| Specific Gravity  <1.005  1.005-1.009  1.010-1.014  1.015-1.019  1.020-1.024  1.025-1.029  >=1.030 | 2942 (5.7%)  8711 (16.7%)  12259 (23.5%)  13572 (26.1%)  9150 (17.6%)  3394 (6.5%)  2030 (3.9%) | <0.0001 |
| Glucose Group  0 (negative, normal, unknown)  1 (1+, 50-150)  2 (2+, 250-1000)  3 (3+, 4+, >500, >1000) | 49054 (94.2%)  1151 (2.2%)  738 (1.4%)  1140 (2.2%) | <0.0001 |
| pH  5.0-5.4  5.5-5.9  6.0-6.4  6.5-6.9  7.0-7.4  7.5-7.9  8.0-8.4  >8.5 | 10812 (20.8%)  12327 (23.7%)  9910 (19.0%)  7862 (15.1%)  6572 (12.6%)  2576 (5.0%)  1567 (3.0%)  443 (0.9%) | <0.0001 |
| Ketones  0 (Negative, Trace)  1(1+, 5-15)  2 (2+20-50)  3 (3+, 4+, >50) | 46332 (89.0%)  3274 (6.3%)  1411 (2.7%)  1065 (2.0%) | <0.0001 |
| Urobilinogen  0 (negative, normal, unknown)  1 (1+, 1-2)  2 (2+, 4)  3 (3+, 8)  4 (4+, 12, >4) | 46872 (90.0%)  3917 (7.5%)  868 (1.7%)  313 (0.60%)  113 (0.21%) | <0.0001 |
| Bilirubin  0 (negative, unknown)  1 (1+, small)  2 (2+, 3+, 4+, large, moderate) | 51329 (98.6%)  507 (0.97%)  247 (0.47%) | <0.0001 |
| Blood  0 (negative, trace, unknown)  1 (*, 1+, small)  2 (**, 2+, moderate)  3 (***, 3+, large) | 42573 (81.7%)  3600 (6.9%)  3397 (6.5%)  2513 (4.8%) | <0.0001 |
| Leukocyte esterase  0 (negative, trace)  1 (*, 1+, small)  2 (**, 2+, moderate)  3 (***, 3+, large) | 41432 (79.6%)  4230 (8.1%)  2912 (5.6%)  3509 (6.7%) | <0.0001 |
| Nitrite  0 (negative, unknown)  1 (positive) | 50138 (96.3%)  1945 (3.7%) | <0.0001 |
| RBC  0 (negative, 0-3, unknown)  1 (4-5)  2 (6-10)  3 (11-20)  4 (21-50)  5 (>50) | 38618 (74.1%)  7106 (13.6%)  1823 (3.5%)  1175 (2.3%)  1095 (2.1%)  2266 (4.4%) | <0.0001 |
| WBC  0 (negative, 0-1,unknown)  1 (2-5)  2 (6-10)  3 (11-20)  4 (21-50)  5 (>50) | 32744 (62.9%)  10102 (19.4%)  2885 (5.5%)  2057 (4.0%)  1869 (3.6%)  2426 (4.7%) | <0.0001 |
| Bacteria  0 (negative, unknown)  1 (few, 1+)  2-3 (moderate, 2+, 3+)  4 (many, 4+) | 37606 (72.2%)  11378 (21.9%)  1741 (3.3%)  1358 (2.6%) | <0.0001 |
| Squamous epithelial cells  0 (none, occas, few, rare, 0-1, unk)  1 (2-5)  2 (6-10)  3 (11-50, moderate)  4 (>50, many) | 34235 (65.7%)  11940 (22.9%)  2185 (4.2%)  2915 (5.6%)  808 (1.6%) | <0.0001 |
