# Supplementary material for: Proteinuria impacts patient survival differentially based on clinical setting: A retrospective cross-sectional analysis of cohorts from a single health system: Retrospective cohort study
Source: Ann Med Surg (Lond). 2019 Aug 1;45:120–6. doi: 10.1016/j.amsu.2019.07.029 (PMC6702410; doi:10.1016/j.amsu.2019.07.029)
Supplement: Multimedia component 3 [file mmc3.docx]

**Table S2.** Six-year overall survival stratified by patient setting and proteinuria group (log rank test).

|  | **Proteinuria Group**  **(Survival %)** | **Proteinuria Group**  **(Survival %)** | **Chi-Square** | **p-Values** |
| --- | --- | --- | --- | --- |
|  |  |  |  | **Tukey-Kramer** |
| **Outpatient Population** |  |  | **198.1** | **<0.0001** |
|  | **A1**  (93.29) | **A2**  (86.10) | 157.1 | <0.0001 |
|  | **A1**  (93.29) | **A3**  (75.00) | 194.9 | <0.0001 |
|  | **A2**  (86.10) | **A3**  (75.00) | 87.6 | <0.0001 |
| **Emergency Population** |  |  | **224.2** | **<0.0001** |
|  | **A1**  (90.46) | **A2**  (85.49) | 86.5 | <0.0001 |
|  | **A1**  (90.46) | **A3**  (67.72) | 178.2 | <0.0001 |
|  | **A2**  (85.49) | **A3**  (67.72) | 8. 8 | 0.0086 |
| **Inpatient Population** |  |  | **132.7** | **<0.0001** |
|  | **A1**  (67.01) | **A2**  (57.97) | 115.2 | <0.0001 |
|  | **A1**  (67.01) | **A3**  (52.36) | 130.3 | <0.0001 |
|  | **A2**  (57.97) | **A3**  (52.36) | 48.2 | <0.0001 |
