# Supplementary material for: Proteinuria impacts patient survival differentially based on clinical setting: A retrospective cross-sectional analysis of cohorts from a single health system: Retrospective cohort study
Source: Ann Med Surg (Lond). 2019 Aug 1;45:120–6. doi: 10.1016/j.amsu.2019.07.029 (PMC6702410; doi:10.1016/j.amsu.2019.07.029)
Supplement: Multimedia component 4 [file mmc4.docx]

**Table S3.** Six-year overall survival stratified by patient setting and proteinuria group (log rank test).

| **Proteinuria Group** | **Patient Group**  **(Survival %)** | **Patient Group**  **(Survival %)** | **Chi-Square** | **p-Values** |
| --- | --- | --- | --- | --- |
|  |  |  |  | **Tukey-Kramer** |
| **A1** |  |  | 4523.1 | **<0.0001** |
|  | **Outpatient**  (93.29) | **Emergency**  (90.46) | 180.2 | <0.0001 |
|  | **Outpatient**  (93.29) | **Inpatient**  (67.01) | 3388.5 | <0.0001 |
|  | **Inpatient**  (67.01) | **Emergency**  (90.46) | 2125.4 | <0.0001 |
| **A2** |  |  | 891.0 | **<0.0001** |
|  | **Outpatient**  (86.10) | **Emergency**  (85.49) | 8.0 | 0.0130 |
|  | **Outpatient**  (86.10) | **Inpatient**  (57.97) | 681.3 | <0.0001 |
|  | **Inpatient**  (57.97) | **Emergency**  (85.49) | 693.4 | <0.0001 |
| **A3** |  |  | 32.2 | **<0.0001** |
|  | **Outpatient**  (75.00) | **Emergency**  (67.72) | 0.03 | 0.9807 |
|  | **Outpatient**  (75.00) | **Inpatient**  (52.36) | 30.3 | <0.0001 |
|  | **Inpatient**  (52.36) | **Emergency**  (67.72) | 21.3 | <0.0001 |
